# The role of KLF2 in regulating hepatic lipogenesis and blood cholesterol homeostasis via the SCAP/SREBP pathway

**DOI:** 10.1016/j.jlr.2023.100472

**Published:** 2023-11-09

**Authors:** Yuhong Huang, Yi Fan Wang, Xiong Zhong Ruan, Chi Wai Lau, Li Wang, Yu Huang

**Affiliations:** 1 Department of Biomedical Sciences, City University of Hong Kong, Hong Kong, China; 2 School of Biomedical Sciences, Chinese University of Hong Kong, Hong Kong, PR China; 3 Shenzhen Research Institute and Li Ka Shing Institute of Health Sciences, Chinese University of Hong Kong, Shenzhen, China; 4 Centre for Lipid Research & Key Laboratory of Molecular Biology for Infectious Diseases (Ministry of Education), Institute for Viral Hepatitis, Department of Infectious Diseases, The Second Affiliated Hospital, Chongqing Medical University, Chongqing, China

**Keywords:** KLF2, SCAP, SREBPs, steatosis, lipogenesis, blood cholesterol

## Abstract

Liver steatosis is a common metabolic disorder resulting from imbalanced lipid metabolism, which involves various processes such as de novo lipogenesis, fatty acid uptake, fatty acid oxidation, and VLDL secretion. In this study, we discovered that KLF2, a transcription factor, plays a crucial role in regulating lipid metabolism in the liver. Overexpression of KLF2 in the liver of *db/db* mice, *C57BL/6J* mice, and *Cd36*-*/*- mice fed on a normal diet resulted in increased lipid content in the liver. Additionally, transgenic mice (*ALB-Klf2*) that overexpressed Klf2 in the liver developed liver steatosis after being fed a normal diet. We found that KLF2 promotes lipogenesis by increasing the expression of *SCAP*, a chaperone that facilitates the activation of SREBP, the master transcription factor for lipogenic gene expression. Our mechanism studies revealed that KLF2 enhances lipogenesis in the liver by binding to the promoter of *SCAP* and increasing the expression of genes involved in fatty acid synthesis. Reduction of *KLF2* expression led to a decrease in *SCAP* expression and a reduction in the expression of SREBP1 target genes involved in lipogenesis. Overexpression of KLF2 also increased the activation of SREBP2 and the mRNA levels of its downstream target *SOAT1*. In *C57BL/6J* mice fed a high-fat diet, overexpression of Klf2 increased blood VLDL secretion, while reducing its expression decreased blood cholesterol levels. Our study emphasizes the novelty that hepatic KLF2 plays a critical role in regulating lipid metabolism through the KLF2/SCAP/SREBPs pathway, which is essential for hepatic lipogenesis and maintaining blood cholesterol homeostasis.

## Graphical Abstract

**Figure:**
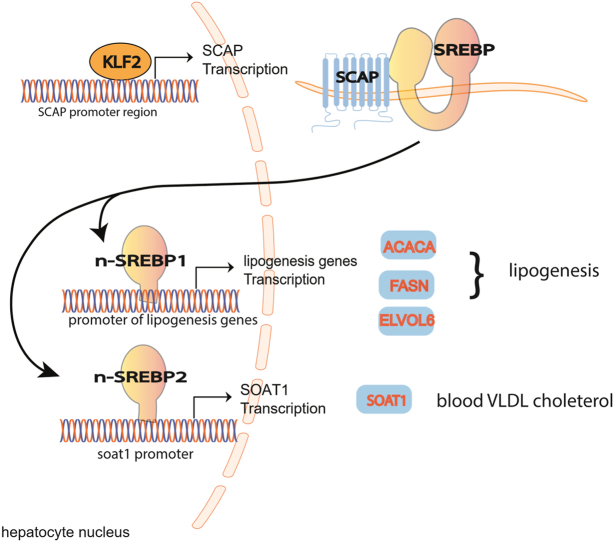

Imbalanced lipid metabolism, including de novo lipogenesis, fatty acid uptake, fatty acid oxidation, and VLDL secretion, can cause liver steatosis, leading to the accumulation of excess fat in the liver (1). De novo lipogenesis is the process by which the liver synthesizes new fats from carbohydrates, while fatty acid uptake refers to the influx of fatty acids from the diet or adipose tissue (2). Elevated levels of de novo lipogenesis are associated with metabolic disorders such as obesity, type 2 diabetes, and nonalcoholic fatty liver disease.

The regulation of hepatic de novo lipogenesis is complex and involves multiple pathways and transcription factors, including SREBPs and ChREBP (3, 4). SREBP is a transcription factor that regulates the expression of genes involved in lipid metabolism, including cholesterol and fatty acid synthesis. SCAP acts as a chaperone for SREBPs, facilitating their activation, and the SREBP-SCAP complex plays a crucial role in the regulation of lipid metabolism and helps maintain cholesterol and fatty acid homeostasis in the body (5, 6, 7, 8).

KLF2, a transcription factor involved in regulating the expression of genes in various tissues and cells, including the liver, has been found to be increased in the liver during liver steatosis (9). While one report suggests that KLF2 may increase fatty acid uptake by upregulation of CD36 (9), the precise role of KLF2 in liver steatosis and blood cholesterol regulation remains unclear. Further research is needed to determine its exact mechanisms of action and the potential of KLF2 as a therapeutic target.

## Materials and Methods

### Cells

To isolate primary hepatocytes from male *C57BL/6J* mice, a two-step collagenase perfusion method was employed (10). The mice were anesthetized and their livers were perfused with 40 ml of perfusion buffer, followed by 15 ml of digestion buffer through the portal vein. The digested liver was then aspirated using a large-bore pipette, and the cells were filtered through a 70 μm cell strainer (Fisher # 07-201-431). After three rounds of centrifugation at 50 *g* for 2 min at 4°C, the primary hepatocytes were purified.

The primary mouse hepatocytes were cultured in Sigma-Aldrich's William's Medium E (W4125) with 10% (v/v) fetal bovine serum (FBS) and 1X antibiotic-antimycotic (Gibco, 15,240,062). Meanwhile, HepG2 (RRID:CVCL_0027), HEK293 A (RRID:CVCL_6910), and HEK293 T cells (RRID:CVCL_0063) were cultured in Dulbecco's modified Eagle's medium (DMEM) with 10% (v/v) FBS and 1X antibiotic-antimycotic (Gibco, 15,240,062). All cells were maintained at 37 °C with 5% CO_2_.

### Animals

A liver-specific promoter and enhancer, the albumin enhancer and promoter (ALB) obtained from pALB-GFP (Addgene, #55759), were used to drive the mouse *Klf2* CDS sequence, which was cloned from a mouse cDNA library. A His tag was fused with *Klf2* at the N-terminus. This construct was supplemented with an SV40 Poly-A signal (plA) from the pGL3 vector (Promega) and inserted into a vector containing a multiple cloning site, pMC.BESPX-MCS2 (an empty vector), MN100B-1, provided by Systembio. Subsequently, the *ALB-Klf2*-plA fragment was excised from the pMC-*ALB-Klf2* plasmid. The resulting linear DNA, *ALB-Klf2*-plA, can be employed for the production of liver-specific transgenic mice.

To generate animal models for the study, *ALB-Klf2*-His tag transgenic mice with a *C57BL/6J* background (RRID:IMSR_JAX:000,664) were created through pronuclear injection with *ALB-Klf2*-His tag linear DNA, Transgenic mice resulting in hepatocyte-specific *Klf2* overexpression were identified through genotyping (genotyping primers are listed in supplemental Table S2). Male *ALB-Klf2*-His tag transgenic mice and wild-type littermate controls were used.

Male total *Cd36* deletion (*Cd3*-*/*-, *C57BL/6J* background) mice were provided by Maria Febbraio (Lerner Research Institute, Cleveland, OH).

For the diet-induced obesity (DIO) mouse model, male *C57BL/6J* mice were fed a high-fat diet (HFD) consisting of 60% kcal fat (Research Diet) for 16 weeks, beginning at 6 weeks of age.

Transient liver-specific *Klf2* overexpression was achieved by tail vein injection of a single dose of Ad-*ALB-Klf2* (1 × 10^∧^9 PFU per mouse) to male *C57BL/6J* mice, *db/db* mice (RRID:IMSR_JAX:000,697) or *Cd3*-*/*- mice fed with normal chow, mice was sacrificed 1 week after adenovirus injection in fed stage.

To achieve knockdown of *Klf2* in DIO male *C57BL/6J* mice (60% fat HFD for 4 months), a single dose of Ad-shRNA *Klf2* (1 × 10^∧^9 PFU per mouse) was administered via tail vein injection.

All mice were provided free access to food and water and were housed under a 12-h light/12-h dark cycle at a temperature of 23°C. All animal experiments were approved by the Animal Experimentation Ethics Committee of the Chinese University of Hong Kong (CUHK) or Chongqing Medical University and were conducted in accordance with institutional guidelines for the humane treatment of laboratory animals.

### AAV production

AAV-CMV-HA-*Klf2* and AAV-CMV-GFP were produced in HEK293FT cells using the polyethyleneimine (PEI) transfection method. A triple transfection of pAAV, pAAV-DJ and pAAV-helper plasmids was performed in 15-cm dishes, as previously described by Gyorgy *et al.* (11). AAVs were extracted from cell lysates and purified by PEG precipitation and applied for discontinuous iodixanol gradient centrifugation at 350,000 *g* for 1 h using OptiPrep™ Density Gradient Medium (D1556, Sigma) (12). OptiPrep™ Density Gradient Medium was removed by 3 times of PBS wash in a Ultracel-100 regenerated cellulose membrane (100 kDa cut off).

### Adenovirus package

To achieve liver-specific overexpression of *Klf2*, a mouse *Klf2* coding sequence with a 6∗His tag was constructed using a hepatocytes-specific albumin enhancer and promoter (ALB) (13). ALB was amplified from plasmid pALB-GFP(addgene, #55759) (13) and cloned into an adenoviral shuttle vector, pAdTrack10, for adenovirus production. An Ad-*ALB-Klf2* adenovirus was generated through recombination in HEK 293A cells using the AdEasy system (14). *Klf2* knockdown was achieved using Ad-shRNA*Klf2* (Vector Biolabs; Cat. No: shADV-213187).

### BODIPY staining

Primary hepatocytes (5000 cells/well) were cultured in 96-well plates with William's medium E supplemented with 10% FBS. After viral infection, cells were incubated with BODIPY Lipid Probes (2 ng/ml, thermos Fisher D3922) for 4 h, then washed three times with PBS. Hoechst 33,342 (2 μM, thermos Fisher 62,249) was used to stain the nuclei for 10 min in assay buffer. Fluorescence was measured using a fluorescence plate reader with excitation at 493 mm and emission at 510 mm for both BODIPY and Hoechst 33,342. The BODIPY signal was normalized to the nuclear signal.

### Oil red O staining

The frozen liver tissues were sectioned and stained with Oil Red O solution (0.5%, Sigma) for visual examination under light microscopy. ImageJ 1.50i (RRID:SCR_003070) was used to calculate the ratio of red area to total tissue area in each microscopic field, which indicates the relative fat content in the liver tissue.

### Western blot

Tissues were homogenized in lysis buffer and centrifuged to obtain cellular proteins. Protein concentration was determined and subjected to SDS-PAGE. Membranes were incubated with various primary antibodies (supplemental Table S1), followed by incubation with a secondary antibody conjugated with horseradish peroxidase. The protein bands were visualized using LumiGLO® Reagent and Peroxide (Cell Signaling, #7003).

### Immunohistochemistry staining

The tissue sections were deparaffinized, rehydrated, and subjected to antigen retrieval using citrate buffer (pH 6.0). After blocking with 5% Normal goat serum (Abcam, Cat# ab7481) in PBST, primary antibodies anti-SREBP1 antibody (Abcam, ab3259) and Rabbit IgG Isotype Control (Thermo Fisher Scientific, Cat# 10500C) were applied and incubated at 4°C overnight. Sections were then washed, incubated with the secondary antibody Goat Anti-Rabbit Immunoglobulins/HRP (Agilent, Cat# P0448), and developed with DAB substrate, followed by counterstaining with hematoxylin. Control sections were also processed to confirm specificity.

### Realtime PCR

To extract total RNA from cells or tissue, the Trizol reagent (Invitrogen Inc.) was used. The High-Capacity cDNA Reverse Transcription Kit (Applied Biosystems) was then employed to carry out reverse transcription, in accordance with the manufacturer's guidelines. For real-time PCR, the QuantiNova SYBR Green PCR Kit (Qiagen) instructions were followed to set up the reaction system. The primer sequences utilized are listed in supplemental Table S2. The reaction conditions consisted of pre-denaturation at 95°C for 2 min, followed by denaturation at 95°C for 5 s, and annealing and extension at 60°C for 20 s, for a total of 40 cycles. Gene expression was evaluated by relative quantitative measurement, whereby 2-ΔΔCt corresponds to the relative expression level of each target gene.

### Dual-luciferase reporter assay

The promoter regions of *SCAP*, *ACACA*, *FASN*, *ELOVL6*, and *SOAT1* were cloned and subcloned into PGL4.10 to generate plasmids for luciferase reporter assay. The cloning primers are listed in supplemental Table S2. The Dual-Luciferase® Reporter Assay System (Promega) was used for dual luciferase assays.

### ChIP-PCR assay

The ChIP assay was conducted on primary hepatocytes following treatment with Ad-*Klf2*-HA tag or Ad-*GFP*. Anti-HA tag antibody (Abcam, ab9110) and the Pierce™ Magnetic ChIP Kit (Thermo Fisher Scientific) were utilized according to the manufacturer's protocol to carry out the ChIP. Real-time PCR was performed to assess the DNA fragment enrichment in the samples obtained from the anti-HA pulldown, whereas the IgG pulldown samples were used as negative controls. The primers used for the ChIP PCR are listed in supplemental Table S2.

### EMSA assay

The LightShift Chemiluminescent EMSA Kit (Thermo Scientific, 20,148) was used to perform EMSA following the manufacturer's instructions. Nuclear extract from HepG2 cells overexpressing *KLF2* was incubated with biotin-labeled DNA probes and unlabeled DNA oligos (supplemental Table S2), as well as KLF2 antibody (EMD Millipore, 09-820), in a reaction buffer consisting of 10 mM Tris, 50 mM KCl, 1 mM DTT, 10 mM ZnCl2, and 50 mg/mlof poly(dI-dC)•poly(dI-dC) (Sigma). Prior to mixing with the other components, an anti-Klf2 antibody was added to the nuclear protein extract and incubated at 4°C for 30 min. The resulting mixtures were loaded onto a 4% polyacrylamide gel and the DNA was transferred to a nylon membrane. The biotin-labeled DNA was then detected by chemiluminescence after crosslinking with UV-light.

### Hepatic VLDL secretion assay

Before the VLDL secretion assay, male *C57BL/6* mice were subjected to a 4-h fast. Baseline blood samples (∼50 μl) are collected from the retro-orbital sinus. For the assay, mice are intraperitoneally injected with poloxamer-407 (P-407) at a dose of 1.0 g/kg of body weight. Blood samples (∼50 μl) are then collected at 1, 2, and 5 h after injection. The mice are kept under isoflurane sedation during the procedure, and they are returned to their cages once bleeding has stopped and anesthesia has been discontinued. Anesthesia and bleeding procedures are repeated for each time point (15). Total cholesterol and HDL-cholesterol levels are determined using enzymatic colorimetric procedures (Biosystems).

### Statistical analysis

To determine the appropriate sample size, a sample size calculator was utilized with a power of α = 0.05 (double-sided) and β = 0.1. The Kruskal-Wallis H test was performed to evaluate whether the samples originated from the same distribution. To ensure unbiased data collection, mice were randomized and investigators were blinded to the data. Results were presented as mean and standard error (means±SEM). Statistical analysis was carried out using GraphPad Prism (RRID:SCR_002798). For blood cholesterol and non-HDL cholesterol data, a two-way repeated measures ANOVA was initially performed, followed by Bonferroni post-tests, and then the area under the curve was calculated and analyzed using either a student *t* test (for two groups) or a one-way ANOVA followed by Bonferroni's multiple comparisons test (for more than two groups). For Western blot and real-time PCR data, a Student's *t* test (for two groups) or a one-way ANOVA followed by Bonferroni's multiple comparisons test (for more than two groups) was utilized for analysis.

## Results

### KLF2 promotes steatosis in mice fed on a normal diet

**Fig. 1:**
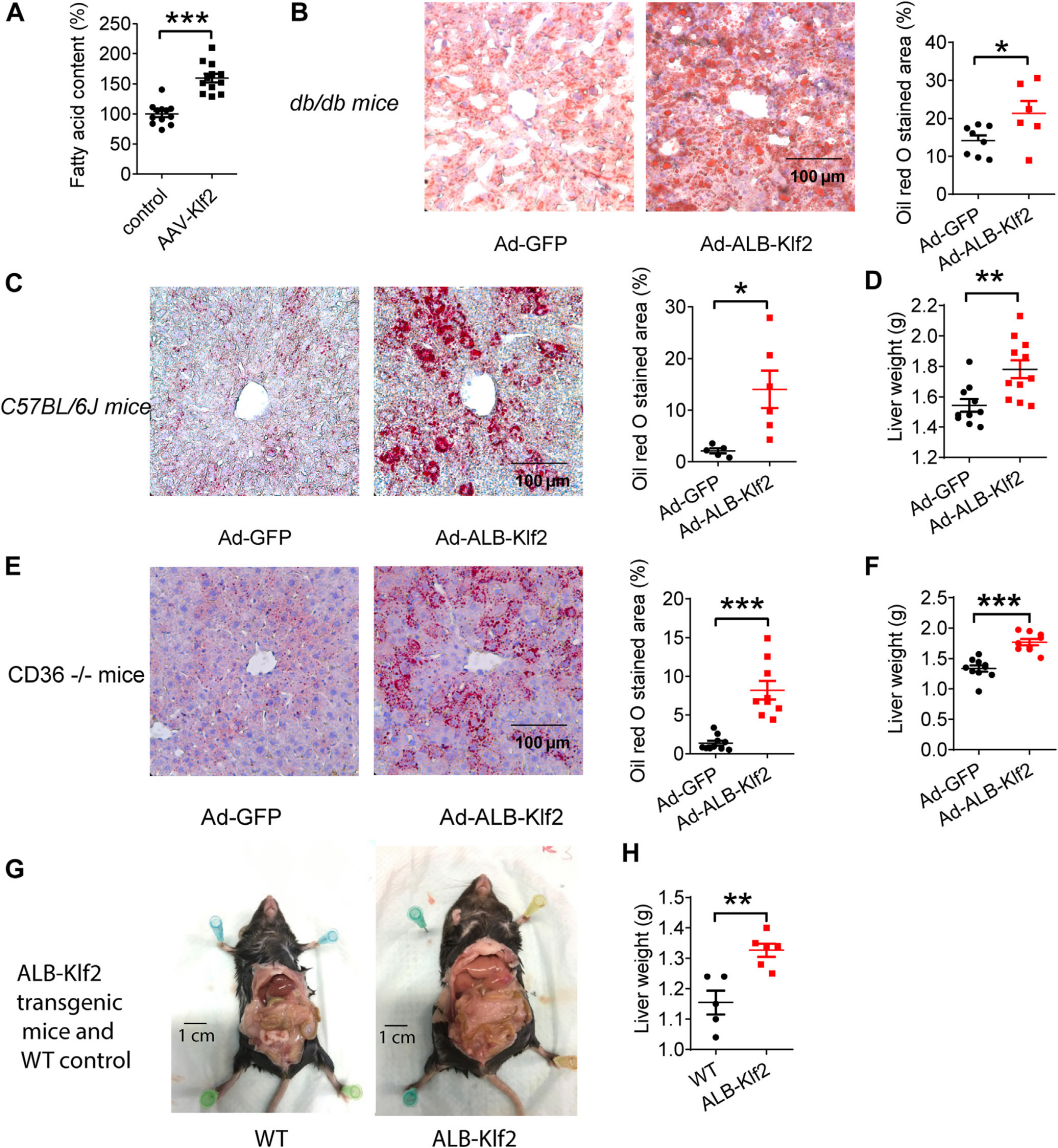
KLF2 promotes steatosis in mice fed with normal chow. A: BODIPY staining of HepG2 cells (n = 12 per group). B: Oil red O staining of liver sections from *db/db* mice fed with normal chow and its statistical analysis (n = 8 for Ad-GFP group, n = 6 for Ad-*ALB-Klf2* group). C: Oil red O staining of liver sections and its statistical analysis (n = 5 for Ad-GFP group, n = 6 for Ad-*ALB-Klf2* group). D: Liver weight of *C57BL/6J* mice fed with normal chow (n = 10 for Ad-GFP group, n = 11 for Ad-*ALB-Klf2* group). E: Oil red O staining of liver sections and its statistical analysis and (F) liver weight of *Cd3*-*/*- mice fed with normal chow (n = 10 for Ad-*GFP* group, n = 9 for Ad-*ALB-Klf2* group). G-H: Representative picture and liver weight of transgenic mice fed with normal chow for 6 months (n = 5 for WT group, n = 6 for *ALB-Klf2* group). Data are presented as mean ± s.e.m. ∗*P* ≤ 0.05, ∗∗*P* ≤ 0.01, ∗∗∗*P* ≤ 0.001.

BODIPY staining indicated an increase in the accumulation of intracellular lipids in HepG2 cells after overexpression of KLF2 with AAV-*Klf2* for 24 h (Fig. 1A). A liver-specific Klf2-overexpressing adenovirus, Ad-ALB-*Klf2*, was generated, in which the *Klf2* CDS sequence was driven by an albumin promoter and enhancer. Oil red O staining of liver sections showed an increase in lipid accumulation in Ad-ALB-*Klf2*-treated *db/db* mice fed a normal diet 1 week after adenovirus injection (Fig. 1B). A similar increase in lipid accumulation was found in *C57BL/6J* mice fed a normal diet in the fed stage after treatment with Ad-ALB-*Klf2* for 1 week, as shown by oil red O staining (Fig. 1C).

**Supplemental Fig. S1:**
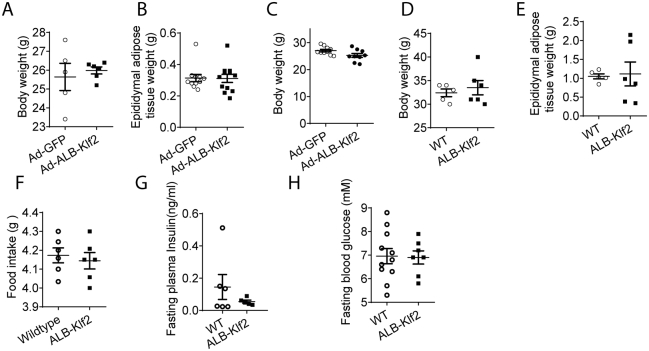
Body weight and adipose tissue weight after Klf2 overexpression A body weight of C57BL/6J mice after virus injection (n = 5 for Ad-GFP group, n = 6 for Ad-ALB-Klf2 group). B: epididymal adipose tissue weight of C57BL/6J mice after virus injection (n = 10 for Ad-GFP group, n = 11 for Ad-ALB-Klf2 group). C: A body weight of CD36-/- mice after virus injection (n = 10 for Ad-GFP group, n = 9 for Ad-ALB-Klf2 group). D and E: body weight and epididymal adipose tissue weight of transgenic mice fed with normal chow for 6 months (n = 5 for WT group, n = 6 for ALB-Klf2 group). F: Food intake of transgenic mice fed with normal chow (n = 6 per group). G: Fasting plasma insulin level of transgenic mice fed with normal diet (n = 6 for WT group, n = 5 for ALB-Klf2 group). H: Fasting blood glucose level of transgenic mice fed with normal chow (n = 11 for WT group, n = 7 for ALB-Klf2 group) Data are mean ± s.e.m.

Additionally, an increase in liver weight was found in another group of *C57BL/6J* mice fed a normal diet in the fed stage and treated with Ad-*ALB-Klf2* for 1 week, but there was no change in body weight and epididymal adipose tissue weight in *Klf2*-overexpressed mice (Fig. 1D and supplemental Fig. S1A, B). Previous reports showed that Klf2 increases liver steatosis by increasing fatty acid uptake via upregulation of *CD36* (9). Therefore, we overexpressed *Klf2* in *C36*^*/−*^ mice fed a normal diet and found that KLF2 induced steatosis as well as an increase in liver weight in *CD36*^*−/−*^ mice in the fed stage (Fig. 1E, F). There was no change in body weight of *CD36*^*−/−*^ mice after *Klf2* overexpression (supplemental Fig. S1C). This suggests that there may be other mechanisms, other that KLF2-induced upregulation of *CD36*, involved in KLF2-induced liver steatosis.

*ALB-Klf2* transgenic mice were generated by transferring into the *ALB-Klf2* sequence into embryos of *C57BL/6J* mice by pronuclear microinjection. Nine-month-old *ALB-Klf2* mice fed a normal diet exhibited evident liver steatosis with an increase in liver weight during the fed stage (Fig. 1G, H). There was no change in body weight and epididymal adipose tissue weight and food intake in normal diet fed *ALB-Klf2* transgenic mice (supplemental Fig. S1, D–F). There was no change in fasting plasma insulin levels (fasted for 16 h) in *ALB-Klf2* mice fed a normal diet when compared to wild-type control (supplemental Fig. S1G). Additionally, 8 h’ fasting blood glucose levels in *ALB-Klf2* mice showed no significant differences when compared to the control group (supplemental Fig. S1H). Thus, we hypothesize that Klf2-induced liver steatosis may occur through an increase in lipogenesis.

### KLF2 promotes liver lipogenesis via SREBP1 activation

**Fig. 2:**
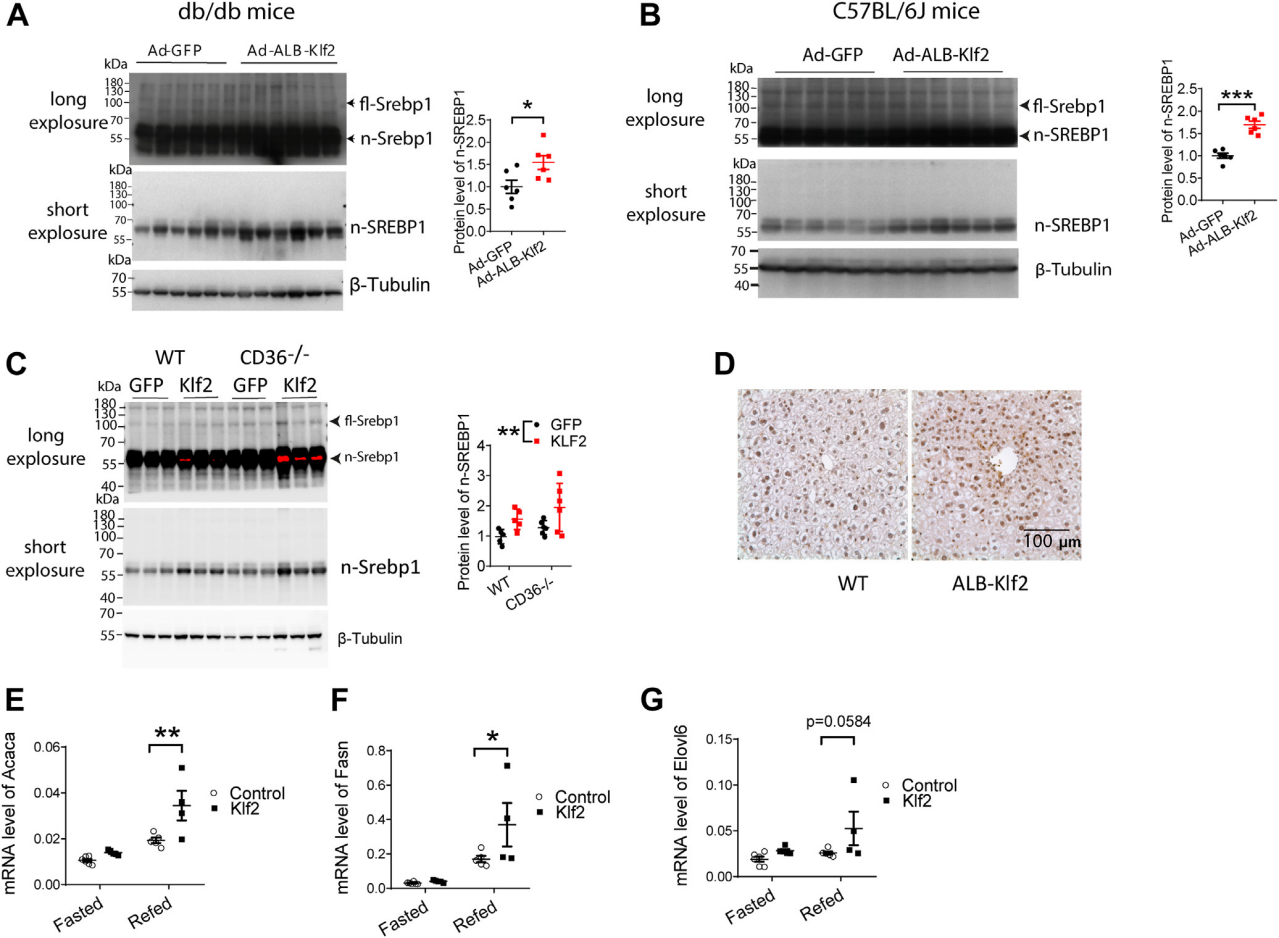
KLF2 increases the protein level of n-SREBP1 and mRNA level of its downstream targets in normal diet-fed mice. A–C: Protein level of n-SREBP1 in liver from *db/db* mice (n = 6 per group), *C57BL/6J* mice (n = 6 per group), and *C*-*/*- mice (n = 5 for WT GFP group and KLF2 group, n = 6 for *CD36-/-* GFP group and KLF2 group). D: IHC staining of SREBP1 in liver sections from *ALB-Klf2* transgenic mice and wildtype control fed with a normal diet for 6 months (n = 4 for WT group and n = 5 for *ALB-Klf2* group). E–G: mRNA level of lipogenesis genes in *C57BL/6J* mice fed with a normal diet (n = 6 for Control group in the fasted stage, n = 5 for Klf2 group in the fasted stage and control group on the refed stage, n = 4 for Klf2 group on the refed stage). Data are presented as mean ± s.e.m. ∗*P* ≤ 0.05, ∗∗*P* ≤ 0.01.

**Supplemental Fig. S2:**
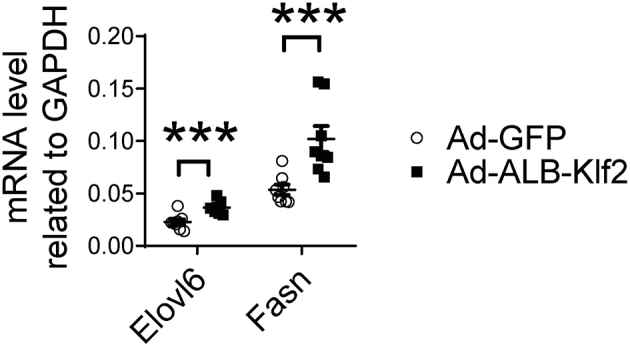
mRNA level of lipogenesis genes in primary hepatocytes (n = 6 per group. Data are mean ± s.e.m. ∗*P* ≤ 0.05, ∗∗*P* ≤ 0.01, ∗∗∗*P* ≤ 0.001).

SREBP1 is an important transcription factor involved in lipogenesis in the liver. We detected the protein levels of full-length SREBP1 and N-terminal SREBP1 (activation form) in the livers of *db/db* mice, *C57BL/6J* mice, and *CD36*^*−/−*^ mice fed a normal diet. We found that KLF2 increased the protein level of N-terminal SREBP1 in the livers of these three mouse models in fed stage 1 week after Ad-*ALB-Klf2* tail vein injection (Fig. 2A–C). Immunobiological staining of liver sections from 9-month-old *ALB-Klf2* transgenic mice fed a normal diet showed that KLF2 increased the nuclear staining of SREBP1, indicating an increase in SREBP1 activation in *ALB-Klf2* transgenic mice (Fig. 2D). The mRNA levels of SREBP1 downstream genes involved in lipogenesis, ACACA, FASN, and ELOVL6, were increased in *Klf2*-overexpressed *C57BL/6J* mice in the refed phase but not in the fasted phase (Fig. 2E–G). In primary hepatocytes treated with Ad-*ALB-Klf2* for 24 h, the genes involved in lipogenesis were also increased (supplemental Fig. S2). These results indicate that Klf2 promotes SREBP1 maturation and increases the expression of its downstream target genes involved in lipogenesis (supplemental Fig. S2).

### KLF2 regulates SCAP expression in hepatocyte and liver

**Fig. 3:**
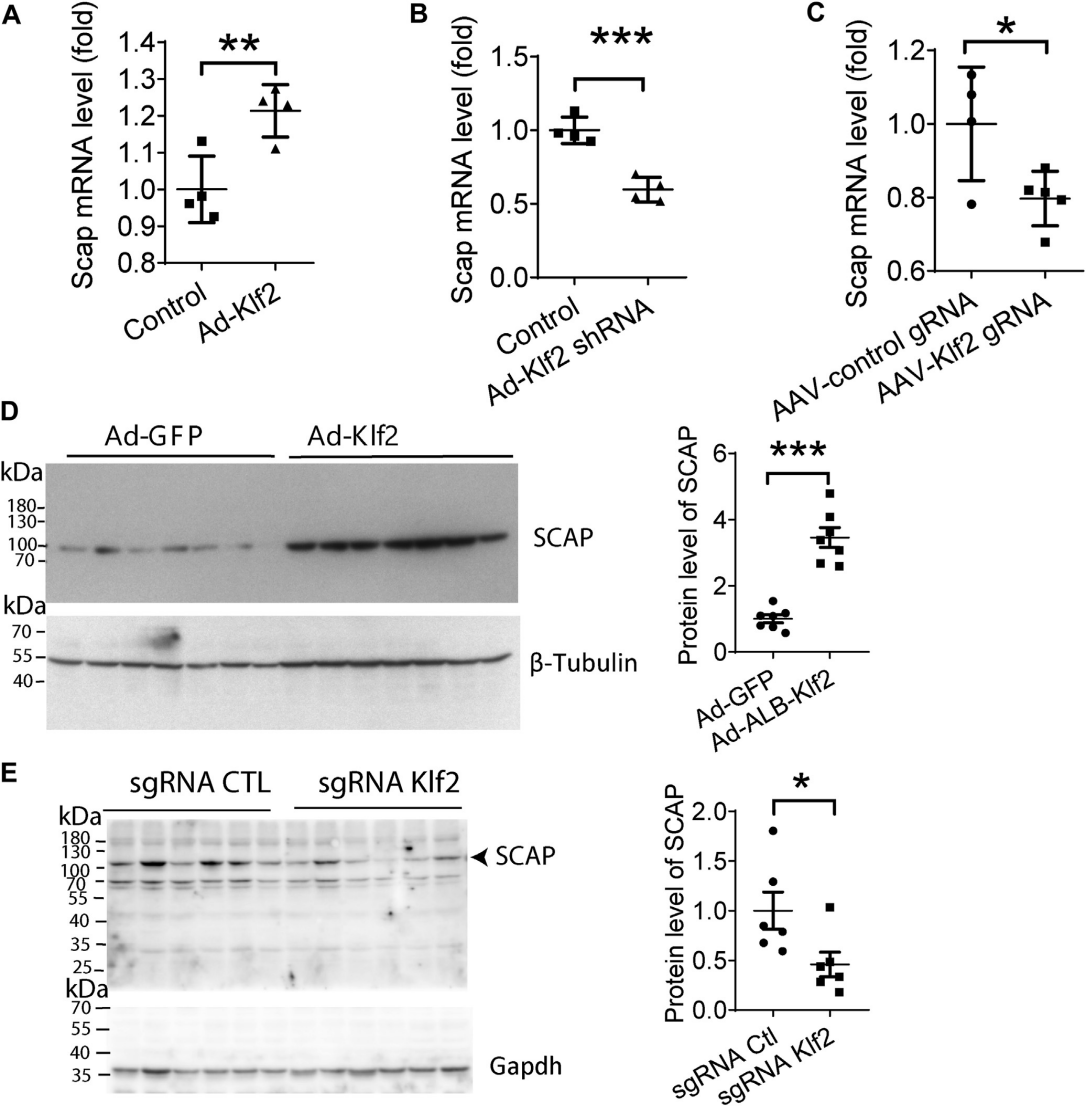
Klf2 upregulates *Scap* expression. A and B: mRNA level of *Scap* in primary hepatocytes (n = 4 per group). C: mRNA level of *Scap* in *C57BL/6J* mice (n = 4 for AAV-control gRNA and n = 5 for AAV-*Klf2* gRNA). D: protein level of SCAP in primary hepatocytes (n = 7 per group). E: protein level of SCAP in liver tissues of *C57BL/6J* mice (n = 6 per group). Data are presented as mean ± s.e.m. ∗*P* ≤ 0.05, ∗∗*P* ≤ 0.01, ∗∗∗*P* ≤ 0.001.

One important chaperone of SREBPs is SCAP. We found that the mRNA level of *Scap* was upregulated in primary hepatocytes after 24 h of KLF2 overexpression using Ad-*ALB-KLF2*. Conversely, the mRNA level of *Scap* was downregulated in primary hepatocytes after 72 h of *KLF2* knockdown using Ad-shRNA *Klf2* (Fig. 3A, B). Liver-specific knockdown of *K*lf*2* by AAV-TBG-Cas9-U6-sgRNA *Klf2* for 1 month also downregulated the *Scap* mRNA level in *C57BL/6J* mice (Fig. 3C). In the presence of insulin in the culture medium, Klf2 increased the protein level of SCAP in primary hepatocytes after 48 h after Ad-*ALB-Klf2* infection (Fig. 3D). Liver-specific knockdown of *Klf2* using AAV-TBG-Cas9-U6-sgRNA *Klf2* for 3 weeks resulted in decreased protein levels of SCAP in the livers of *db/db* mice compared to AAV-TBG-Cas9-U6-sgRNA Ctl-treated control mice (Fig. 3E).

### KLF2 regulates lipogenesis by binding to the SCAP promoter region and activating SREBP1 downstream targets

**Fig. 4:**
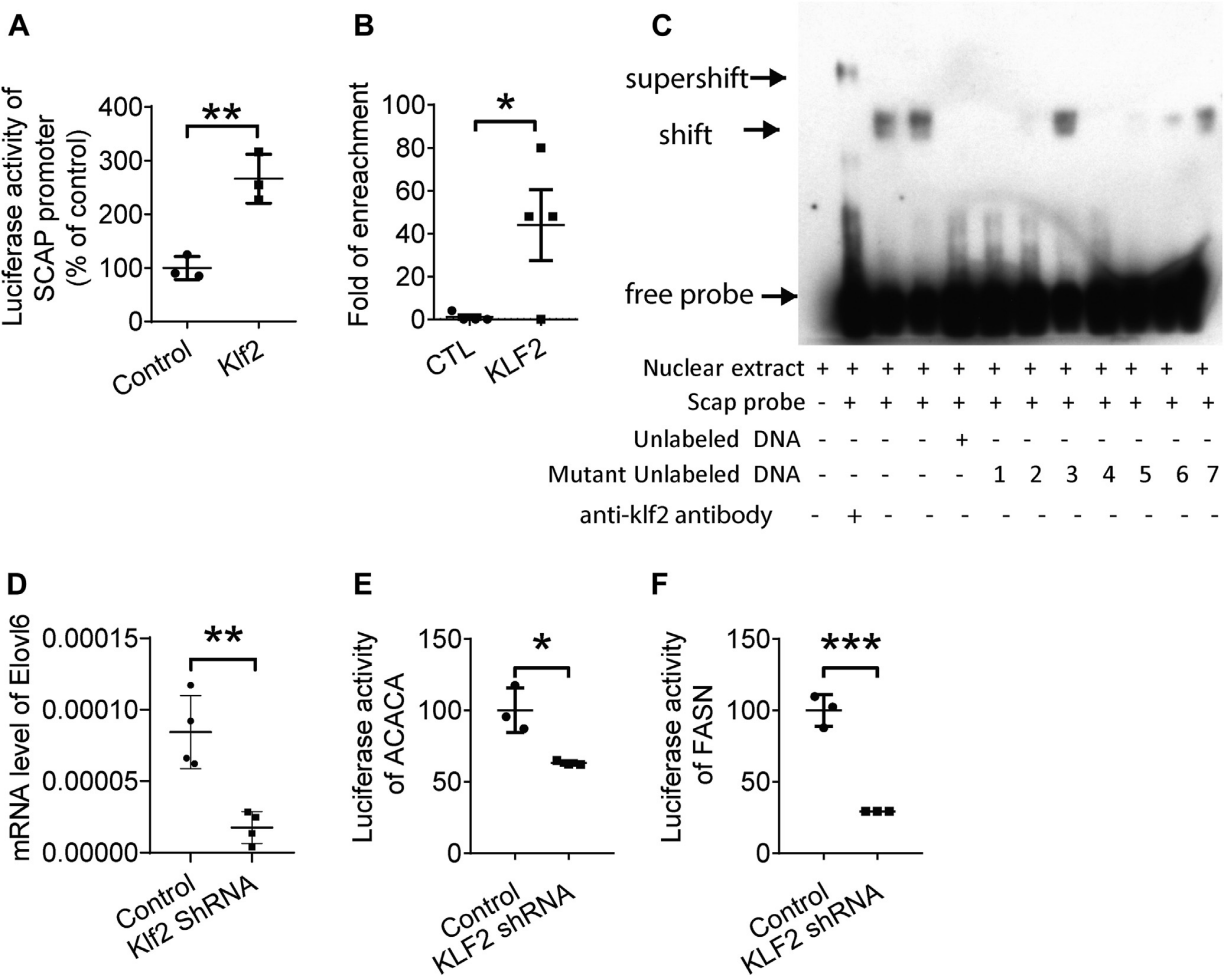
KLF2 transcriptionally regulates SCAP by binding to its promoter. A: Reporter gene assay detecting SCAP promoter activity in primary hepatocytes (n = 3 per group). B: Chip-PCR detects enrichment of DNA region of SCAP promoter after anti-HA antibody pulldown in Ad-*Klf2*-HA tag overexpressed primary hepatocyte (n = 4 per group). C: EMSA assay detecting KLF2 binding to the promoter sequence of SCAP (n = 4 for SCAP probe binding to nuclear extract with or without unlabelled DNA, n = 2 for nuclear extract binding to SCAP probe and KLF2 antibody, n = 1 for each mutant unlabelled DNA binding). D: mRNA level after KLF2 knockdown in primary hepatocytes (n = 4 per group). E and F: reporter gene assays of lipogenesis genes after KLF2 knockdown in HepG2 cells (n = 4 per group). Data are mean ± s.e.m. ∗*P* ≤ 0.05, ∗∗*P* ≤ 0.01, ∗∗∗*P* ≤ 0.001.

**Supplemental Fig. S3:**
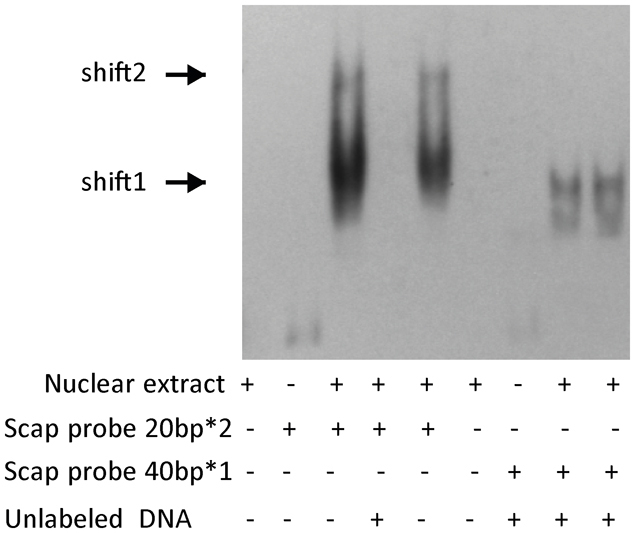
EMSA of SCAP probe in KLF2 over expressed primary hepatocytes nuclear extract (n = 2 per group).

Since KLF2 regulates both mRNA level and protein level of SCAP, we hypothesize that KLF2 might transcriptional regulate SCAP. Luciferase reporter assay showed that KLF2 increases SCAP promoter activity in HepG2 cells after Ad-ALB-*Klf2* treated for 24 h (Fig. 4A). CHIP-PCR analysis showed there was significant enrichment of SCAP promoter DNA region after anti-HA antibody pulldown in Ad-*Klf2*-HA treated primary hepatocytes (Fig. 4B). EMSA assay indicated that KLF2 binds to the *SCAP* DNA probe from the promoter region of *SCAP*, and this binding can be competitively inhibited by unlabeled DNA. Additionally, a mutation on the KLF2 binding site reverses the competitive inhibition, indicating the presence of a KLF2 binding site in this region (Fig. 4C). We generated two additional probes with the same length (40 bp), one containing two binding sites (20 bp∗2) and another containing one binding site (40 bp∗1). Two shifted bands were observed in the 20 bp∗2 probe, while only one shift was observed in the 40 bp∗1 probe (supplemental Fig. S3). These results further confirmed the KLF2 binding sequence.

Since SCAP facilitates the activation of SREBP1, the knockdown of KLF2 might decrease SREBP1 activation. Indeed, SREBP1 downstream targets that regulate lipogenesis were downregulated after *KLF2* knockdown. The mRNA levels of *Elovl6* were downregulated after being treated with Ad-shRNA *Klf2* for 72 h. Additionally, the promoter activities of *ACACA* and *FASN* were downregulated in vitro after KLF2 knockdown using shRNA *Klf2* for 72 h in HepG2 cells (Fig. 4D–F).

### KLF2 regulates cholesterol metabolism through SOAT by modulating SREBP2 activation

**Fig. 5:**
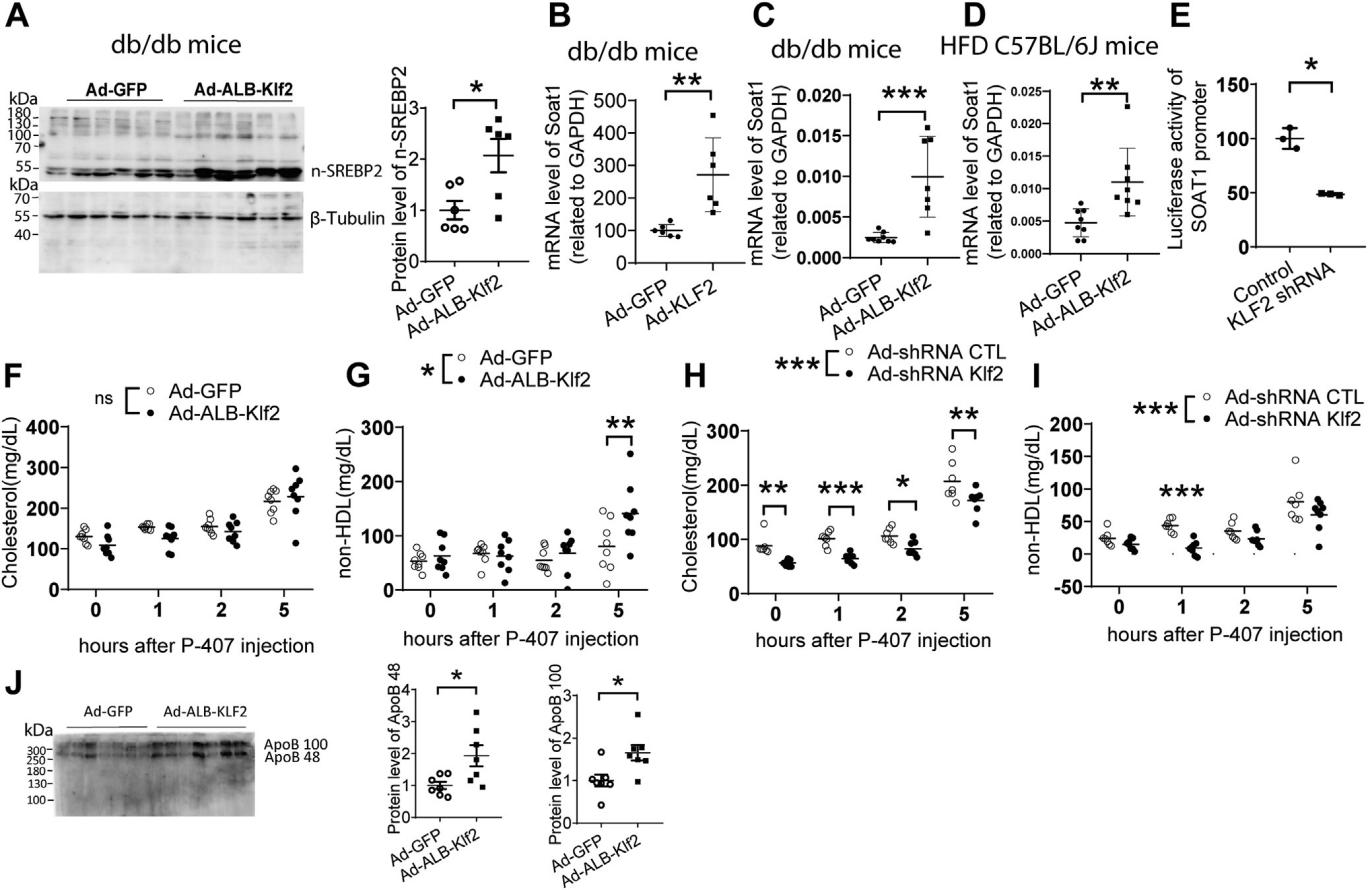
KLF2 regulates blood cholesterol via SREBP2/SOAT1 pathway. A: protein level of SREBP2 in liver from *db/db* mice (n = 6 per group). B and C: SOAT1 mRNA level in liver from *db/db* mice after treatment with Ad-*Klf2* (n = 6 per group) or Ad-*ALB-Klf2* (n = 8 for Ad-GFP group and n = 7 for Ad-*ALB-Klf2* group). D: SOAT1 mRNA level in liver from *C57BL/6J* mice fed with a high-fat diet after treatment with Ad-*ALB-Klf2* (n = 8 per group). E: Reporter assay of SOAT1 after Klf2 knockdown in HepG2 cells (n = 3 per group). F–I: Blood lipid profile after Poloxamer 407 injection in *C57BL/6J* mice fed with a high-fat diet (n = 8 for Ad-*GFP*, Ad-*ALB-Klf2*, Ad-shRNA *Klf2* group, n = 7 for Ad-shRNA CTL group). J: ApoB protein level from 10 μl plasma. Data are mean ± s.e.m. ∗*P* ≤ 0.05, ∗∗*P* ≤ 0.01, ∗∗∗*P* ≤ 0.001.

**Supplemental Fig. S4:**
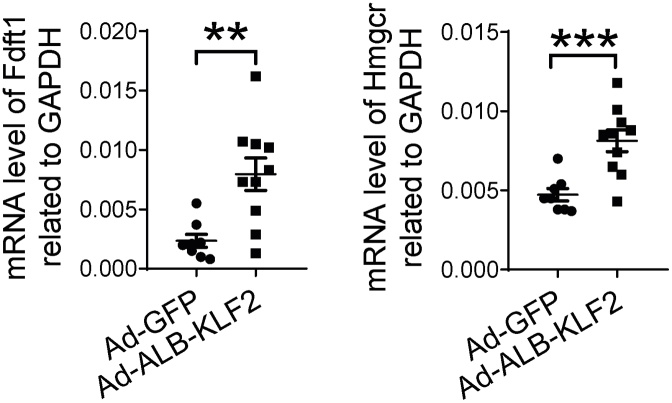
Real-time PCR data detected the mRNA levels in livers from diet-induced obese C57BL/6J mice (n = 8 per group. Data are mean ± s.e.m. ∗*P* ≤ 0.05, ∗∗*P* ≤ 0.01, ∗∗∗*P* ≤ 0.001).

Since SCAP also facilitates SREBP2 activation, we detected protein levels in primary hepatocytes after KLF2 overexpression. We found that KLF2 increases the N-terminal of SREBP2 in livers from *db/db* mice treated with Ad-*ALB-Klf2* for 1 week (Fig. 5A). mRNA levels of SOAT1, the downstream target of SREBP2, were upregulated in global KLF2 overexpression induced by Ad-*Klf2* for 1 week in *db/db* mice as well as in liver-specific Klf2 overexpression achieved by Ad-ALB-Klf2 for 1 week in *db/db* mice and *C57BL/6J* mice fed with a high-fat diet (Fig. 5B–D). Other SREBP2 downstream targets, such as Farnesyl-diphosphate farnesyltransferase 1 (FDFT1) and 3-hydroxy-3-methylglutaryl-CoA reductase (HMGCR), were also upregulated by KLF2 in the liver of *C57BL/6J* mice fed a high-fat diet after being treated with Ad-*ALB-Klf2* for 1 week (supplemental Fig. S4). Reporter assays showed that SOAT1 promoter activity was downregulated in HepG2 cells after *KLF2* knockdown (Fig. 5E). SOAT is an enzyme that catalyzes the transfer of a fatty acyl group from a fatty acyl-CoA molecule to the hydroxyl group of cholesterol, forming cholesteryl esters. Inhibition of SOAT has been shown to lower plasma cholesterol levels and prevent atherosclerosis (16, 17). Liver-specific overexpression of *KLF2* induced by Ad-*ALB-Klf2* for 1 week in *C57BL/6J* mice fed a high-fat diet for 3 months did not change the overall blood cholesterol level. However, an increase in VLDL cholesterol level was indicated by the rise in plasma non-HDL cholesterol after treatment with poloxamer 407, which is used to block LDL uptake. Conversely, KLF2 knockdown achieved by Ad-shRNA *Klf2* for 1 week decreased plasma cholesterol levels and VLDL secretion in *C57BL/6J* mice fed a high-fat diet for 3 months (Fig. 5F–I). KLF2 increased the levels of ApoB100 protein in the plasma of *C57BL/6J* mice fed a high-fat diet after Ad-ALB-*Klf2* treatment for 1 week, further confirming the role of KLF2 in increasing VLDL secretion (Fig. 5J).

## Discussion

The present study demonstrates that KLF2 functions as a key regulator of lipid metabolism in the liver. KLF2 promotes lipogenesis by increasing the expression of SCAP, thereby facilitating the activation of SREBP1, the master transcription factor responsible for the expression of lipogenic genes. Our results reveal that overexpressing of KLF2 in the liver of mice led to an increase in intracellular lipid accumulation and liver weight. Conversely, a reduction in KLF2 expression led to a decrease in SCAP expression and a reduction in the expression of SREBP1 target genes involved in lipogenesis.

Furthermore, our investigation highlights the critical role of KLF2 in maintaining blood cholesterol homeostasis. Overexpression of KLF2 in *C57BL/6J* mice fed a high-fat diet increased blood VLDL secretion while reducing KLF2 expression corresponds with reduced blood cholesterol levels.

The precise mechanisms underlying how KLF2 regulates lipid metabolism and blood cholesterol homeostasis remain incompletely understood. Previous research suggests that KLF2 may increase fatty acid uptake by upregulating *CD36* (9). The expression of KLF2 is heightened in the livers of both ob/ob mice and wild-type mice fed a high-fat diet (HFD). The introduction of KLF2 through adenovirus-mediated overexpression in healthy mice amplifies hepatic steatosis by increasing the expression of the *Cd36* gene. Conversely, the suppression of KLF2 using shRNA-based techniques reduces liver weight and attenuates triglyceride accumulation in ob/ob mice, with this impact being dependent on the presence of *CD36*. Comprehensive investigations encompassing chromatin immunoprecipitation and site-directed mutagenesis unequivocally establish CD36 as a direct transcriptional target of KLF2 (9).

However, our study has found that KLF2 promotes liver steatosis by increasing lipogenesis through activation of the SREBP1 pathway. The present results indicate that KLF2 induces an increase in lipid accumulation and liver weight in mice fed a normal diet, and this effect is independent of *CD36* expression. We also generated *ALB-Klf2* transgenic mice, which developed liver steatosis after being fed a normal diet for 6 months. Immunobiological staining of liver sections from *ALB-Klf2* transgenic mice showed an increase in nuclear SREBP1 staining, indicating an increase in SREBP1 activation. Furthermore, mRNA levels of SREBP1 downstream genes involved in lipogenesis, such as ACACA, FASN, and ELOVL6, were found to be increased in KLF2-overexpressed mice.

A previous study by Chen *et al.* (9) reported that *KLF2* knockdown improved steatosis in ob/ob mice fed a normal diet. Our study provides evidence that KLF2-induced lipogenesis may underlie the underlying mechanism for this observed effect.

In addition to its role in liver lipogenesis, our study also investigated the impact of KLF2 on cholesterol metabolism. We found that KLF2 promotes the maturation of SREBP2 and enhances the expression of its downstream target SOAT1, which is responsible for esterifying cholesterol in the liver. This esterification of cholesterol is a critical step in the assembly and secretion of VLDL particles, which transport cholesterol to other parts of the body.

SOAT1 is expressed in most cells, and although global inhibition of SOAT1 decreases blood cholesterol levels, it does not rescue atherosclerosis. This may be due to a deficiency of SOAT1 in macrophages, leading to the accumulation of free cholesterol (18). However, practical inhibition of SOAT1 or liver-specific inhibition of SOAT1 has been shown to prevent atherosclerosis (16, 17). In our study, we found that hepatic KLF2 regulates SOAT1, providing a new pathway for regulating blood cholesterol without affecting macrophage SOAT1.

Interestingly, although KLF2 increased SCAP expression, overexpression of KLF2 in the livers of mice did not result in an overall increase in blood cholesterol levels. This observation may be due to the fact that SREBP2 activity is regulated by several other factors, including cellular cholesterol and lipid levels. When cellular cholesterol levels are low, SREBP2 is activated through a process that involves SCAP and Insig, a cholesterol-sensing protein. Conversely, when cellular cholesterol levels are high, cholesterol molecules bind to Insig, which then binds to SCAP, preventing SREBP2 activation and leading to decreased cholesterol synthesis. Other lipid molecules, such as oxysterols and fatty acids, can also affect SREBP2 activation (1, 5, 6).

The present study also reveals that KLF2 upregulates genes associated with lipogenesis during the refeeding phase, but not during fasting. This difference is likely due to the reduced activity of SREBP1 during fasting, caused by decreased insulin signaling and activation of AMP-activated protein kinase (AMPK) in response to low energy levels. AMPK phosphorylates SREBP1, resulting in decreased transcriptional activity and a subsequent reduction in the expression of lipogenic genes (19, 20).

In summary, our study provides evidence that the KLF2/SCAP/SREBPs pathway plays a crucial role in lipid metabolism in the liver. Our findings indicate that KLF2 promotes steatosis by upregulating SCAP expression and activating SREBP1 downstream targets that are involved in lipogenesis. Additionally, KLF2 plays a role in regulating blood cholesterol homeostasis by activating SREBP2 and increasing its downstream target SOAT1. Our results suggest that this pathway has implications for metabolic disorders and could be a potential target for therapeutic interventions.

## Data Availability

All data are available in the main text or the supplementary materials.

## Supplemental data

This article contains supplemental data.

## Conflict of interest

The authors declare that they have no conflicts of interest with the contents of this article.
