# Supplementary material for: The role of KLF2 in regulating hepatic lipogenesis and blood cholesterol homeostasis via the SCAP/SREBP pathway
Source: J Lipid Res. 2023 Nov 9;65(1):100472. doi: 10.1016/j.jlr.2023.100472 (PMC10805670; doi:10.1016/j.jlr.2023.100472)
Supplement: Supplemental Table [file mmc1.docx]

**Table S1**. Primary antibodies for western blot

| Names of antibody | Brands | Cat. No. |
| --- | --- | --- |
| anti-SCAP antibody | Abcam | ab91323 |
| anti-SREBP1 antibody | Abcam | ab3259 |
| anti-KLF2 antibody | EMD Millipore | 09-820 |
| anti-GAPDH antibody | Cell signaling technology | 5174 |
| anti-β-tubulin antibody | Santa cruz | sc-9104 |
| anti-SREBP2 antibody | Abcam | ab30682 |
| anti-ApoB antibody | Novus Biologicals | NOU-NB200-527 |

**Table S2.** Primers or oligo DNA

|  | gene | sequence |
| --- | --- | --- |
| Genotyping ALB-KLF2 | ALB-KLF2 GT F | TCCAATCCCATGGAGAGGATGA |
|  | ALB-KLF2 GT B | AGGAACCAATGAAATGCGAGGT |
| Genotyping WT | ALB-KLF2 GT F | TCCAATCCCATGGAGAGGATGA |
|  | ALB-KLF2 GT C | CAGGGACTGAGGACACGC |
| Real-time  PCR primers | Acaca | ATGGGCGGAATGGTCTCTTTC |
|  |  | TGGGGACCTTGTCTTCATCAT |
|  | Fasn | GGAGGTGGTGATAGCCGGTAT |
|  |  | TGGGTAATCCATAGAGCCCAG |
|  | Elovl6 | GAAAAGCAGTTCAACGAGAACG |
|  |  | AGATGCCGACCACCAAAGATA |
|  | Scap | GCTCGTGACCACCTACATCAT |
|  |  | CCCCACTTGGACTTGACCA |
|  | Soat1 | GAAACCGGCTGTCAAAATCTGG |
|  |  | TGTGACCATTTCTGTATGTGTCC |
| Reporter assay cloning primers | SCAP promoter | ATCAGTGGTACCTTGGGGGTGAGCAAGAGTG |
|  |  | ATCAGTCTCGAGCCGCGGTCCGGTGTT |
|  | ACACA promoter | ACTTAGGTACC GTTGGGACCCCTTCCAGTTATTATT |
|  |  | ATCTCTAAGCTT AGTTTCTCCAGGTCCCCGGT |
|  | FASN promoter | actatggtaccTCCAACATCACGTGGACAGAG |
|  |  | acgtgaagctt TAGTGCAGACGGTGACAGTGAA |
|  | SOAT1 promoter | ACTTAGGTACC TGATTATGAAGTCACGAGGGC |
|  |  | ACTATAGATCT CACCACCGTTACCTGAGGC |
| Chip-PCR primers | Scap promoter | GCAGTGCCAGACTACGCAT |
|  |  | GCGGTCCGGTGTTTGGA |
| EMSA probes | SCAP probe 20*1 | GAGGGGAAAGGTAGGAGTTG |
|  |  | CAACTCCTACCTTTCCCCTC |
|  | SCAP probe 20*2 | GAGGGGAAAGGTAGGAGTTGGAGGGGAAAGGTAGGAGTTG |
|  |  | CAACTCCTACCTTTCCCCTCCAACTCCTACCTTTCCCCTC |
|  | SCAP probe 40*1 | GGCGGAGCGGGAGGGGAAAGGTAGGAGTTGAGAGGTGAAG |
|  |  | CTTCACCTCTCAACTCCTACCTTTCCCCTCCCGCTCCGCC |
|  | SCAP probe 30*1 | AGCGGGAGGGGAAAGGTAGGAGTTGAGAGG |
|  |  | CCTCTCAACTCCTACCTTTCCCCTCCCGCT |
|  | SCAP mutant oligo 1 | AGCGGTGAGGGAAAGGTAGGAGTTGAGAGG |
|  |  | CCTCTCAACTCCTACCTTTCCCTCACCGCT |
|  | SCAP mutant oligo 2 | AGCGGGAGTTTAAAGGTAGGAGTTGAGAGG |
|  |  | CCTCTCAACTCCTACCTTTAAACTCCCGCT |
|  | SCAP mutant oligo 3 | AGCGGGAGGGGCCCGGTAGGAGTTGAGAGG |
|  |  | CCTCTCAACTCCTACCGGGCCCCTCCCGCT |
|  | SCAP mutant oligo 4 | AGCGGGAGGGGAAATTCAGGAGTTGAGAGG |
|  |  | CCTCTCAACTCCTAGAATTCCCCTCCCGCT |
|  | SCAP mutant oligo 5 | AGCGGGAGGGGAAAGGTTAAAGTTGAGAGG |
|  |  | CCTCTCAACTTTAACCTTTCCCCTCCCGCT |
|  | SCAP mutant oligo 6 | AGCGGGAGGGGAAAGGTAGGCAGTGAGAGG |
|  |  | CCTCTCACTGCCTACCTTTCCCCTCCCGCT |
|  | SCAP mutant oligo 7 | AGCGGGAGGGGAAAGGTAGGAGTCATGAGG |
|  |  | CCTCATGACTCCTACCTTTCCCCTCCCGCT |
